# The *Pseudomonas aeruginosa* effector protein TesG regulates alternative activation of macrophages through NLRC5

**DOI:** 10.1128/msphere.00681-25

**Published:** 2025-10-31

**Authors:** Qianhua Zhang, Yige Zhang, Ruihuan Wang, Kailun Wang, Teng Ma, Chaoyu Zou, Yongxin Zhang, Xueli Hu, Huan Liu, Jing Sherly Li, Yang Yang, Zhuochong Liu, Miao Tang, Yilin Liu, Hongliang Li, Yu Tang, Jing Li, Xikun Zhou

**Affiliations:** 1 Department of Biotherapy, Cancer Center and State Key Laboratory of Biotherapy, West China Hospital, Sichuan University 670149, Chengdu, China; 2 State Key Laboratory of Oral Diseases, National Clinical Research Center for Oral Diseases, Chinese Academy of Medical Sciences Research Unit of Oral Carcinogenesis and Management, West China Hospital of Stomatology, Sichuan University 168305, Chengdu, China; Vanderbilt University Medical Center, Nashville, Tennessee, USA

**Keywords:** TesG, NLRC5, macrophage polarization, infection immunity

## Abstract

*Pseudomonas aeruginosa* is one of the most common pathogenic bacteria in the clinic. Its large genome and strong genetic plasticity enable it to survive in various environments, posing a significant threat to patient health. We previously identified a key effector protein, TesG, secreted by the type I secretion system, which plays a crucial role in the chronic infection process of *P. aeruginosa*. However, the underlying mechanism remains incompletely understood. In this study, we newly discovered that TesG can induce alternative activation of macrophages and explored its mechanisms through a series of *in vivo* and *in vitro* experiments. We found that TesG promotes the expression of the NLRC5 protein, thereby inducing the polarization of macrophages toward the M2 phenotype. The activation of macrophages induced by TesG primarily occurs through the currently known mechanisms of NLRC5. These findings suggest that *P. aeruginosa* can regulate the alternative activation of macrophages through the TesG/NLRC5 signaling pathway during chronic infection, significantly aiding its evasion of the host immune system killing. In summary, our data highlight the complex infection strategies developed by pathogenic bacteria to achieve chronic infection.

**IMPORTANCE:**

During the transition from acute to chronic *Pseudomonas aeruginosa* infection, bacteria modulate the host’s immune microenvironment to evade immune responses, ensuring long-term survival. Clinical studies have confirmed that the effector protein TesG (secreted by a type I secretion system) can serve as a potential clinical biomarker for chronic *P. aeruginosa* lung infections. Our findings indicate that TesG promotes the alternative activation of macrophages through the regulation of NLRC5, thereby suppressing inflammatory responses and facilitating the progression of chronic pulmonary infections. These discoveries enhance our understanding of the complex interplay between *P. aeruginosa* and the host, laying the groundwork for developing precise diagnostic and therapeutic strategies targeting chronic pulmonary infections.

## INTRODUCTION

*Pseudomonas aeruginosa* is a gram-negative opportunistic pathogen that commonly infects immunocompromised individuals, including patients with severe burns, urinary tract infections, AIDS, chronic obstructive pulmonary disease, bronchiectasis, and cystic fibrosis (1–3). The pathogenic potential of *P. aeruginosa* is closely related to its expression of a large number of virulence factors, which can not only break through host immune defenses in infections but also gain advantageous ecological niches in interspecies competition with other microorganisms (4, 5). The adaptive nature of pathogen infection can lead to the eventual transformation of acute *P. aeruginosa* infections into chronic infections at specific sites in the body, such as the lungs. In chronic infection, the immune microenvironment at the site of host infection is aberrantly regulated, and neither the host immune system nor interventional therapies are effective in clearing the pathogen (6). In chronic infection, *P. aeruginosa* undergoes a series of phenotypic changes to adapt to the lung environment, including virulence factors, motility, iron acquisition, and antibiotic resistance (7–11). Therefore, an in-depth study on the pathogenic mechanisms of *P. aeruginosa* is expected to provide new insights for developing novel antibacterial treatment, which holds great academic value and clinical importance.

Our previous investigations have identified a new effector protein of the type I secretion system called TesG (T1SS effector protein targeting small GTPases), which plays a significant role in the chronic *P. aeruginosa* infection (12). Remarkably, TesG exhibits distinct expression patterns compared to acute-phase virulence factors, with marked upregulation specifically during chronic infection. By suppressing RhoGEF-Rho GTPase signaling, TesG impairs neutrophil recruitment, macrophage function, and cytokine production, enabling *P. aeruginosa* to evade host immunity while sustaining pathogenicity. This finding suggested that TesG may have distinct functions from other virulence factors and may play an irreplaceable role in immunoregulation during *P. aeruginosa* chronic infection. In addition, a recent clinical study based on our preliminary data indicates that TesG expression may serve as a promising biomarker for chronic *P. aeruginosa* lung biofilm infections, highlighting the importance of further investigating its role in these infections (13).

In our previous work, we revealed the immune cell atlas in acute and chronic lung infection models of *P. aeruginosa* using single-cell sequencing. We found that, although the proportions and numbers of immune cells did not change significantly in chronic infection compared to uninfected conditions, the interactions between cells increased significantly in chronic infection, suggesting that immune cells, such as macrophages, are in an abnormally activated state (14). Macrophages are an important innate immune population that maintains homeostasis and defends against pathogens. Their phenotype and function are regulated by the microenvironment, exhibiting a high degree of plasticity (15, 16). Macrophages typically exist in different transcriptional states depending on the environment or conditions, with the well-known M1 phenotype (M1-type, classical activation) and M2 phenotype (M2-type, alternative activation) representing the two extremes. When initial pathogen infection occurs, macrophages first exhibit the M1-type, releasing proinflammatory cytokines to activate the immune system and eliminate the pathogen (17–19). However, to prevent excessive inflammatory responses from causing tissue damage, the body later regulates the conversion of macrophages to the M2-type (alternative activation), secreting anti-inflammatory cytokines to suppress inflammation. M2-type macrophages contribute to tissue repair, remodeling, angiogenesis, and maintenance of stability (20–22). Our previous findings showed that TesG modulates cell viability and cytokine secretion in macrophages, which may be critical for *P. aeruginosa* evasion of host immune killing. However, how TesG regulates macrophage functions remains to be elucidated.

The nucleotide-binding oligomerization structural domain NOD-like receptor (NLR) is a major component of the cytoplasmic innate immune-sensing pathway (23, 24). NLRC5 is a member of the NLR family, primarily expressed in immune cells (25), and is widely involved in the development of various pathological processes, such as renal fibrosis and renal diabetes mellitus (26–28). However, the role of the *Nlrc5* gene in immunity remains unclear. Some studies demonstrated that the overexpression of NLRC5 in cells leads to enhanced IFN-α expression (29, 30), while the knockdown of endogenous *Nlrc5* in THP-1 cells results in decreased Sendai virus-mediated type I interferon expression (31). NLRC5 can also function as a critical NLR sensor that responds to PAMP/DAMP and DAMP/cytokine combinations to drive innate immune cell death (32, 33). However, studies on the mechanism underlying the role of NLRC5 in bacterial immune regulation are somewhat limited.

In this study, we found that TesG treatment induced the polarization of macrophages toward alternative activation. Further investigation revealed that the regulation of macrophage activation by TesG depends on the expression of NLRC5. This finding emphasizes the immune regulatory mechanisms involving NLRs, particularly NLRC5, during chronic infection by *P. aeruginosa*. Therefore, we anticipate that this study will significantly contribute to a deeper understanding of *P. aeruginosa* strategies for evading host immunity.

## RESULTS

### TesG induces the alternative activation of macrophages *in vitro*

Our previous studies have shown that TesG competitively inhibits the activity of eukaryotic small GTPase in macrophages, thereby suppressing host inflammation and promoting the development of chronic lung infections (12). To further characterize the immunomodulatory function of TesG during *P. aeruginosa* infection, we established an experimental platform using two well-characterized macrophage models: iBMDMs and MH-S cells.

**Fig 1:**
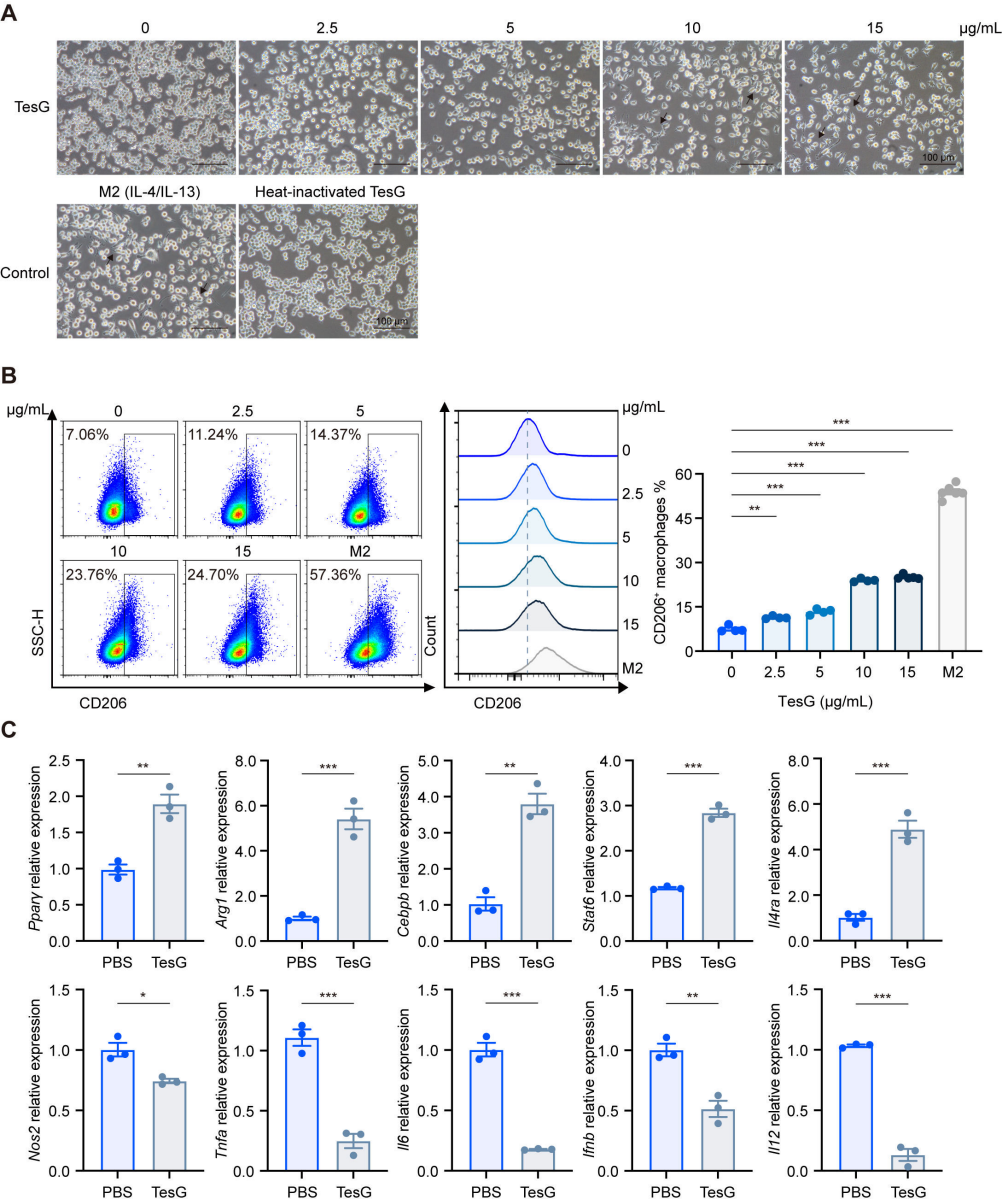
TesG induces the polarization of macrophages *in vitro*. iBMDMs were incubated overnight with purified TesG protein (0–15 μg/mL) or controls. (**A**) Representative phase-contrast images. Arrows indicate cells with M2-like morphology. M2 positive control: IL-4/IL-13 (20 ng/mL each); heat-inactivated TesG control: 10 µg/mL. Scale bar, 100 µm. (**B**) Flow cytometric assessment of M2 polarization. Overlay histogram shows CD206 expression profiles in iBMDMs, and the bar chart quantifies the proportion of CD206^+^ cells (*n* = 4–6). (**C**) The mRNA expression of macrophage polarization-related genes in iBMDMs treated overnight with TesG (10 µg/mL) or PBS. Data points represent biological replicates within individual experiments; bar graphs show mean ± SEM. Similar results were observed in three independent experiments. *P < 0.05; **P < 0.01; ***P < 0.001; and ns, not significant (**B**: one-way ANOVA with Tukey’s multiple comparison test; **C**: two-tailed unpaired Student’s *t* test).

Following overnight exposure to purified TesG, iBMDMs exhibited a dose-dependent increase in cell spreading and elongation, shifting from rounded to spindle-like morphologies consistent with M2 polarization (Fig. 1A). In contrast, heat-inactivated TesG resembled baseline, whereas IL-4/IL-13 produced the expected M2-like appearance. To test whether TesG directly modulates macrophage polarization at the cellular level, we analyzed the M2 marker (CD206) expression by flow cytometry following treatment with increasing TesG concentrations (0–15 μg/mL) (Fig. S1A). Quantitative assessment revealed that TesG induced an increase of CD206 marker expression in iBMDMs, and this trend became more evident as the concentration of TesG increased (Fig. 1B). Similar results were also observed in TesG-treated MH-S cells (Fig. S1B). Consistently, RT-qPCR analysis revealed differential expression of macrophage polarization-related genes upon TesG treatment (Fig. 1C; Fig. S1C). These findings demonstrate that TesG induces M2-type polarization of macrophages *in vitro*.

### TesG induces polarization of macrophages *in vivo*

**Fig 2:**
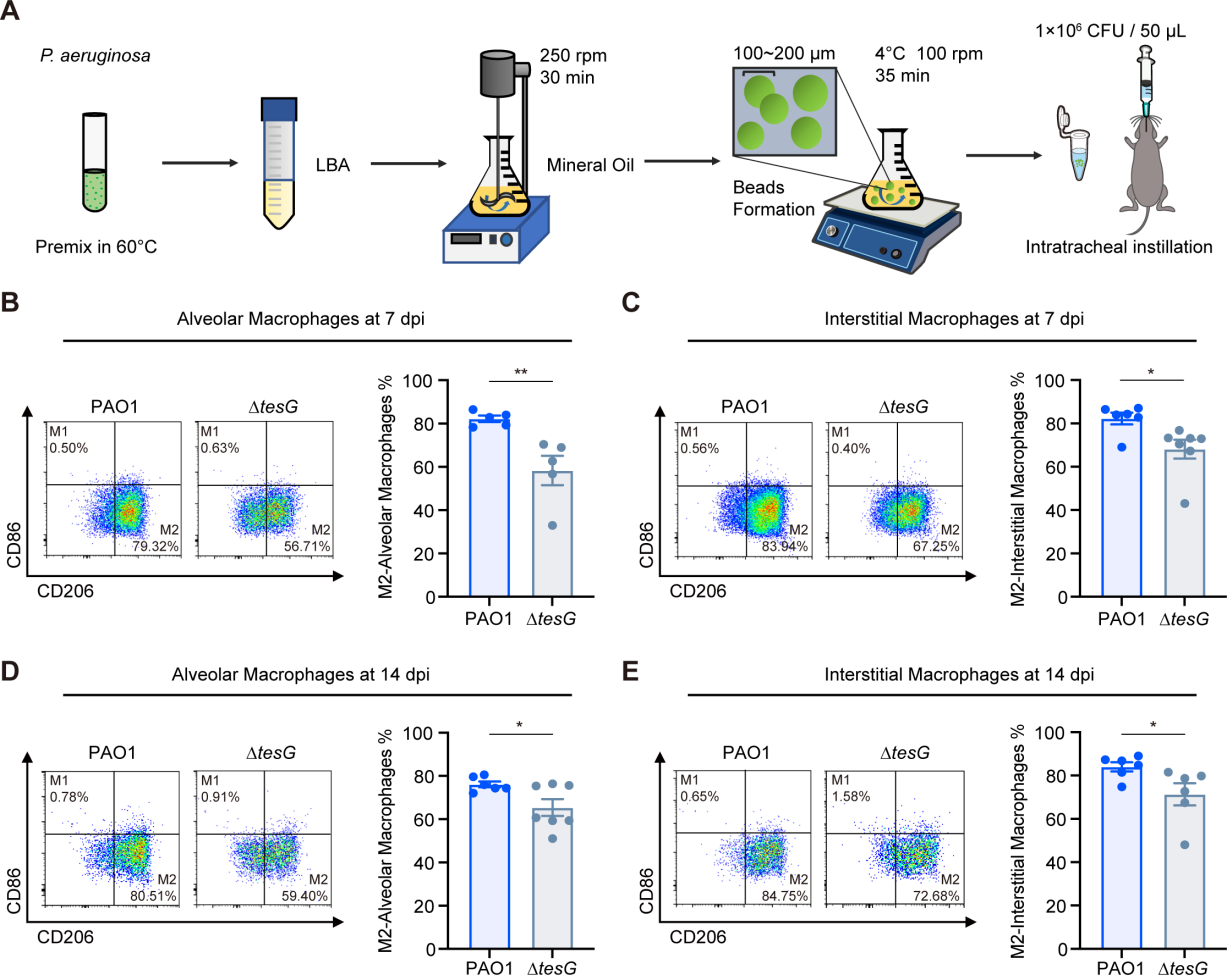
TesG induces the polarization of macrophages *in vivo*. (**A**) Constructed mouse model of chronic lung infection. Chronic lung infections were modeled by tracheal injection of 50 µL of agarose bead-encapsulated wild-type PAO1 (1 × 10^6^ CFU) or ∆*tesG* mutant strains. Mouse lung tissues were collected at 7 and 14 days post-treatment, and single-cell suspensions were prepared for macrophage detection by flow cytometry. (**B and C**) Polarization of AM and IM at 7 days post-infection. (**D and E**) Macrophage polarization profiling at 14 days post-infection. Data are from one representative experiment with five to seven mice per group; points represent individual mice, bars show mean ± SEM. Similar results were observed in two independent experiments. Unpaired *t*-test: *P < 0.05; **P < 0.01.

To assess whether TesG can also induce the M2 polarization of macrophages *in vivo*, we established chronic lung infection models in C57BL/6 mice using agarose bead-encapsulated PAO1 wild-type and ∆*tesG* mutant strains (12, 34) (Fig. 2A). Based on our previous demonstration that the expression of TesG is specifically upregulated during chronic infection (peaking at days 7–18), we analyzed lung tissues at days 7 and 14 post-infection. Flow cytometry was used to quantify polarization states in two important macrophage types: alveolar macrophages (AMs) and interstitial macrophages (IMs) (Fig. S2A). At 7 days post-infection (7 dpi), the proportion of AMs polarized to the M2 phenotype was significantly greater in the PAO1 group than in the ∆*tesG* group (Fig. 2B), whereas there was no significant difference in M1-polarized macrophages (Fig. S2B). Similarly, the proportion of M2-polarized IMs was significantly increased in the PAO1 group than in the ∆*tesG* group (Fig. 2C). In both AMs and IMs on day 14 of infection, the proportion of M2-polarized cells in the PAO1 group remained greater than that in the ∆*tesG* group (Fig. 2D and E). There was also no significant difference in M1-polarized macrophages (Fig. S2C). Thus, these results indicated that TesG can induce M2-type macrophage polarization during chronic *P. aeruginosa* infection.

### TesG-induced macrophage polarization was only weakly affected by known targets of TesG

Our previous work identified small GTPase family proteins (e.g., RhoA and Rac2) as TesG-interacting targets by pulldown-mass spectrometry (12). To determine whether TesG acts through the RhoA axis, we transfected iBMDMs with an siRNA targeting RhoA and evaluated TesG-induced macrophage polarization by flow cytometry (Fig. S3A). It showed that the siRhoA had a subtle but reproducible effect on TesG-induced macrophage alternative activation (Fig. S3B). We then examined additional small GTPase candidates and confirmed by co-immunoprecipitation that TesG interacts with RhoA, Rac2, Rab7, Grb2, Gnai2, and Fcer1g (Fig. S3C). Similarly, siRNAs for these proteins were synthesized (Fig. S3A), and TesG treatment was performed to detect macrophage polarization. The results showed that silencing these immune-interacting proteins had no significant effect on the TesG-induced macrophage alternative activation (Fig. S3D). It suggests that the TesG-induced alternative activation is largely independent of small GTPase-mediated regulation but may rely on other regulatory targets.

### TesG induces macrophage polarization via NLRC5

We next explored the underlying mechanism by which TesG regulates macrophage polarization. Following intravenous administration of TesG protein for three doses, we harvested mouse lung tissues at 24 h post-final injection for whole-transcriptome microarray analysis. The Gene Ontology enrichment analysis of differentially expressed genes revealed significant enrichment of immune response pathways (Fig. S4A). Among these differentially expressed immune-related genes, NLR family member NLRC5 caught our attention (Fig. S4B). NLRC5 is typically regarded as a negative regulator of innate immunity that can suppress the onset of inflammatory responses (33, 35, 36). This finding is consistent with the observation that TesG can induce macrophage polarization toward the M2-type, thereby inhibiting the initiation of inflammation.

**Fig 3:**
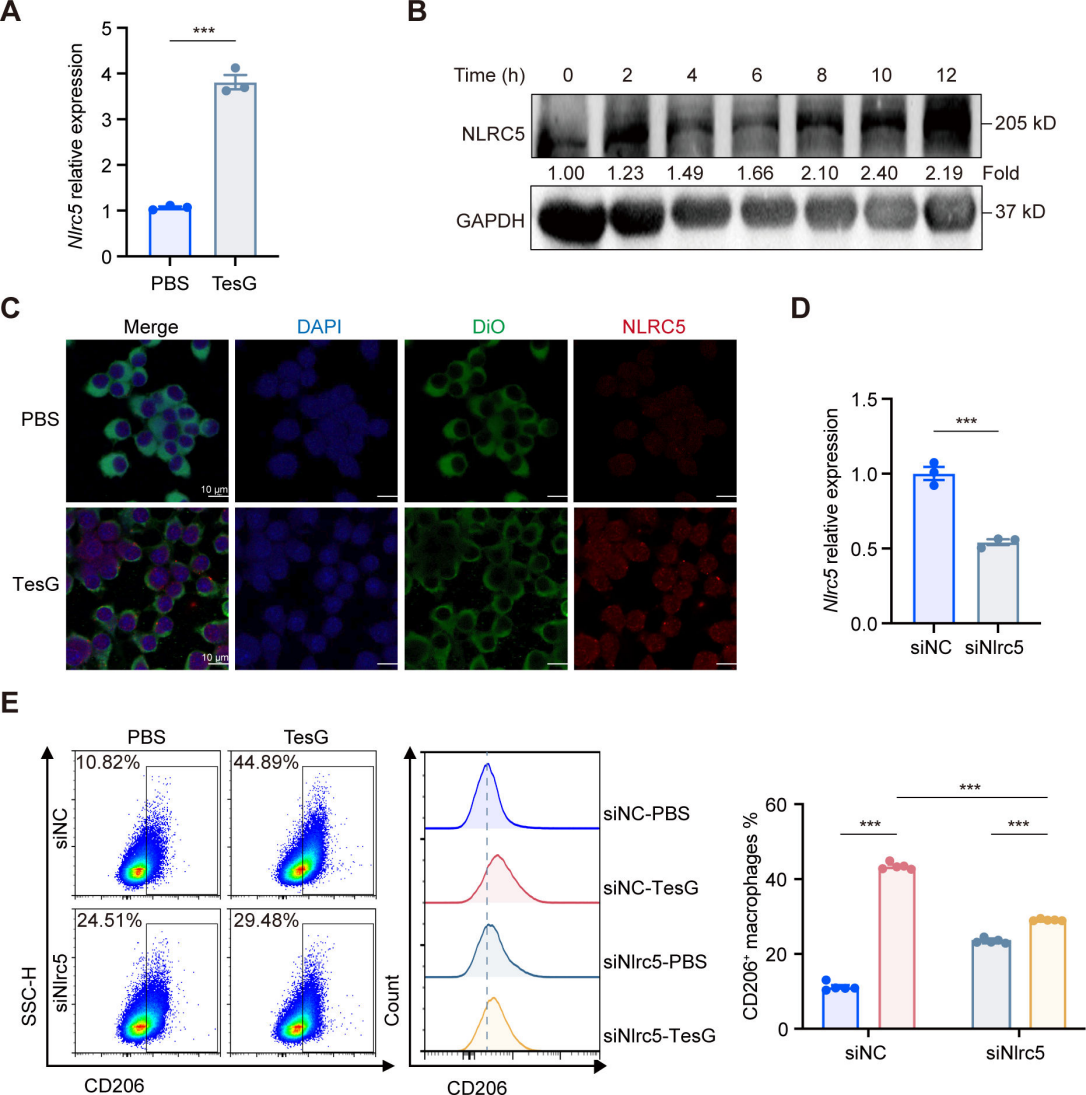
TesG induces the polarization of macrophages via NLRC5. (**A**) The mRNA expression level of *Nlrc5* was upregulated in iBMDMs following overnight treatment with 10 µg/mL TesG protein. (**B**) Western blot analysis demonstrates that TesG progressively induced NLRC5 protein expression. Protein band intensities and mRNA relative expression levels were quantified as the ratio of the indicated protein or mRNA to GAPDH. These values were then normalized to the average expression levels in the PBS-treated or TesG 0 h-treated group, which were set to 1.00. (**C**) Immunofluorescence staining shows TesG-induced NLRC5 (red) expression in iBMDMs. Membranes were stained with DiO (green) and nuclei with DAPI (blue). Scale bar: 10 µm. (**D**) Interference efficiency of *Nlrc5* siRNA in iBMDMs. (**E**) Flow cytometry analysis of macrophage polarization following TesG treatment in *Nlrc5*-knockdown iBMDMs. All experiments included three to five biological replicates; all data are presented as the mean ± SEM. Statistical significance: *P < 0.05; **P < 0.01; ***P < 0.001; and ns, not significant (**A and D**: two-tailed unpaired Student’s *t* test; E: two-way ANOVA with Tukey’s multiple comparison test).

To verify whether NLRC5 is involved in macrophage polarization, we treated macrophages with TesG protein overnight and detected the mRNA expression of *Nlrc5*. Consistent with the microarray results, the mRNA expression of *Nlrc5* was upregulated in iBMDMs after TesG treatment (Fig. 3A). We further explored whether TesG affects the expression of NLRC5 protein. Similarly, cells treated with TesG protein were collected at various time points for Western blot analysis of NLRC5 protein expression dynamics. Notably, NLRC5 protein levels in TesG-treated iBMDMs showed progressive accumulation during the first 12 h of treatment (Fig. 3B). This result was also visualized by immunofluorescence (Fig. 3C). The above results confirmed that TesG increased the mRNA and protein levels of NLRC5 in iBMDMs. Moreover, we transiently interfered with the expression of the *Nlrc5* gene using siRNA (Fig. 3D) and stably knocked it down using shRNA (Fig. S4C), with the cells subsequently treated with TesG protein overnight. The flow cytometry results of both transient interference and stable knockdown of *Nlrc5* gene expression showed a decreased degree of TesG-induced iBMDM polarization toward the M2-type (Fig. 3E; Fig. S4D), and the results were consistent with those observed in MH-S cells (Fig. S5A through D). The above results indicate that NLRC5 is a key mediator of TesG-induced M2 polarization.

### TesG does not directly interact with NLRC5

Many NOD family proteins, including NLRC5, can form complexes that act as pattern recognition receptors for pathogens (30, 37). We speculated that NLRC5 might recognize TesG. To test this, Flag-tagged TesG and Myc-tagged murine and human NLRC5 overexpression vectors were constructed (Fig. S6A and B). There is no direct interaction between TesG and NLRC5 protein by co-immunoprecipitation (Co-IP) (Fig. S6C and D). Previous reports have indicated that NLRC4’s recognition of pathogen patterns depends on NAIPs (38). To investigate whether NAIPs mediate the interaction between TesG and NLRC5, we cotransfected 293T cells with TesG, NLRC5, and NAIPs overexpression plasmids and detected the interactions of these proteins by Co-IP (Fig. S6E and F). The results showed that TesG still did not interact with NLRC5 in the presence of NAIPs. We further hypothesized that endogenous NLRC5 in macrophages might directly recognize TesG. To test this, purified TesG was co-incubated with macrophage lysates, and endogenous NLRC5 was pulled down. However, no interaction between these proteins was detected in either experimental approach (Fig. S6G). Taken together, these results suggest that the regulatory function of TesG on macrophage polarization through NLRC5 is not mediated by direct interaction between the two proteins.

### TesG regulates macrophage polarization through known regulatory mechanisms of NLRC5

**Fig 4:**
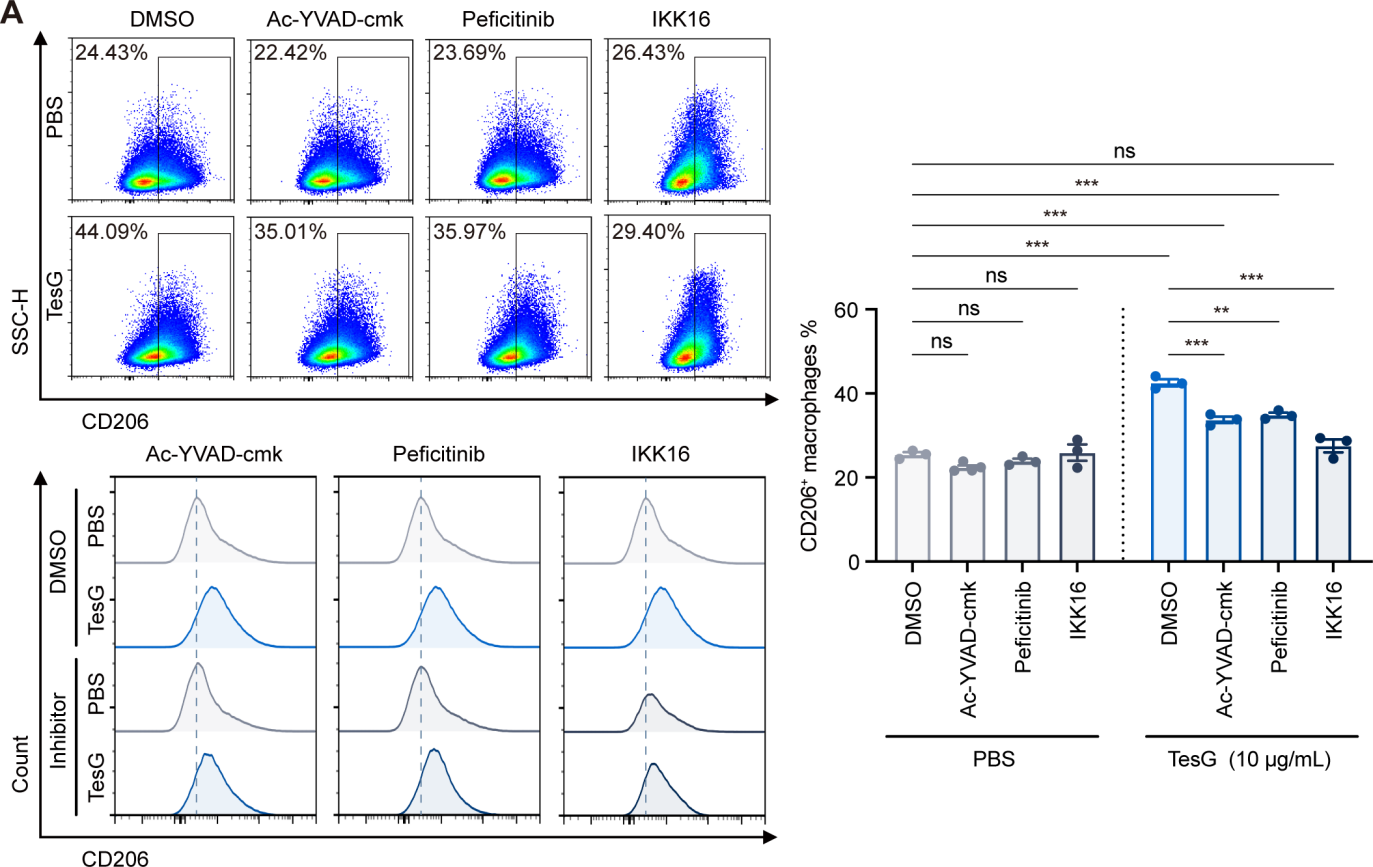
TesG regulates the polarization of macrophages through known functions of NLRC5. (**A**) iBMDMs were treated overnight with either TesG protein (10 µg/mL) alone or in combination with NLRC5 pathway inhibitors (5 µM Ac-YVAD-cmk, caspase-1 inhibitor; 1 µM peficitinib, JAK inhibitor; and 0.1 µM IKK16, IKK-2 inhibitor), and macrophage polarization was analyzed by flow cytometry. Data are presented as mean ± SEM of three to four biological replicates, with statistical significance determined by one-way ANOVA with Tukey’s multiple comparison test: *P < 0.05; **P < 0.01; ***P < 0.001; and ns, not significant.

The main known regulatory mechanisms of NLRC5 involve the caspase-1, NF-κB, and JAK pathways (33, 35, 39, 40). To further investigate whether these known mechanisms of NLRC5 regulation affect TesG-induced NLRC5-mediated regulation of macrophage polarization, we employed specific inhibitors for caspase-1 (Ac-YVAD-cmk), NF-κB (IKK16), and JAK (peficitinib) during TesG treatment. Flow cytometry analysis revealed that the TesG-induced increase in CD206 expression was partially attenuated by these specific pathway inhibitors (Fig. 4A). Under TesG stimulation, NLRC5 might modulate the activity of caspase-1, NF-κB, and JAK pathways to influence polarization. Over-inhibition of these pathways appears to disrupt this process, leading to reduced M2 marker induction. These findings indicate that TesG drives macrophage polarization at least partly through the typical immunomodulatory functions of NLRC5.

### TesG/NLRC5 signaling regulates macrophage polarization *in vivo*

**Fig 5:**
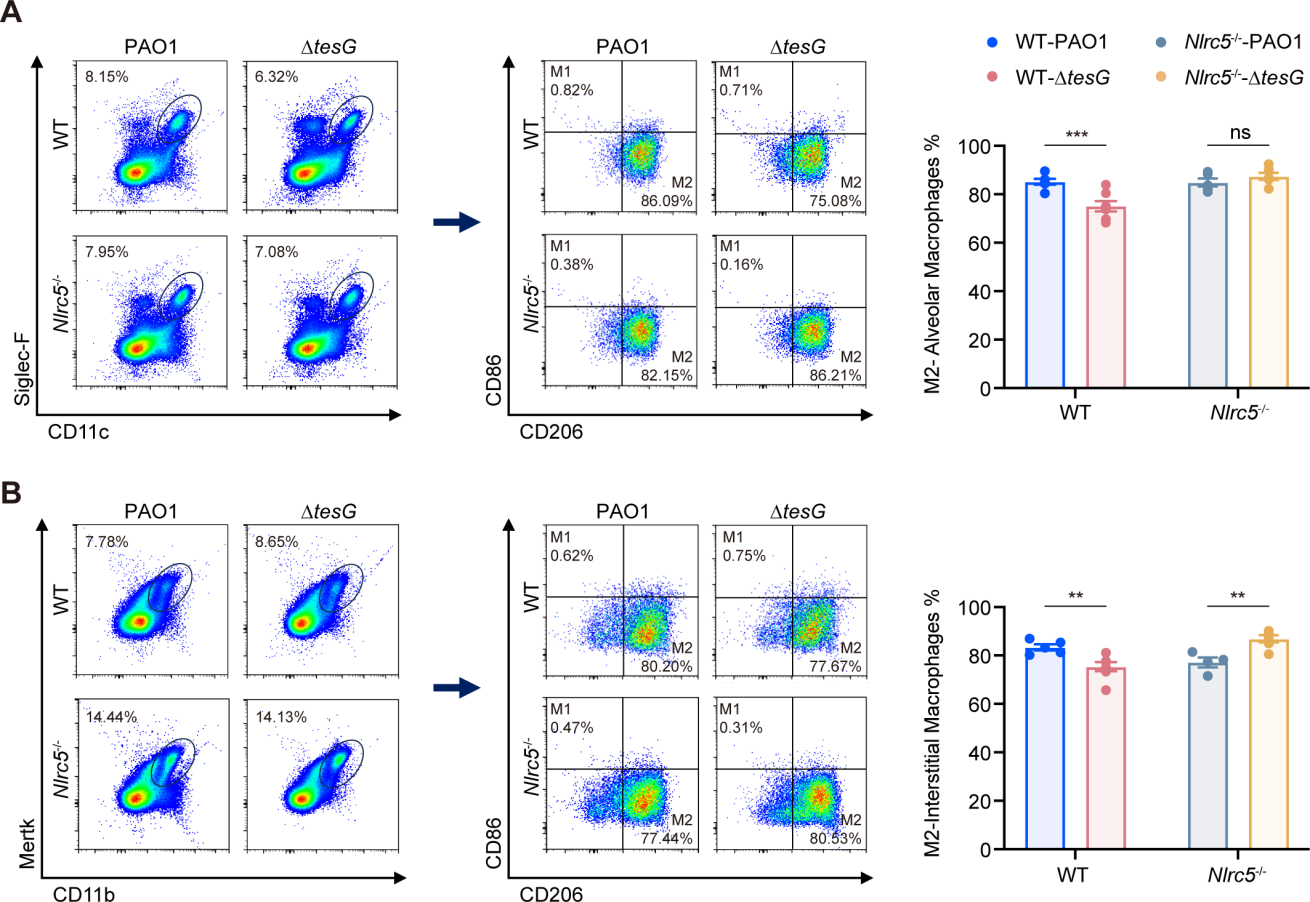
TesG/NLRC5 signaling regulates the polarization of macrophages *in vivo*. Construction of chronic infection models with PAO1 and ∆*tesG* strains in both C57BL/6 WT and *Nlrc5* knockout mice. (**A**) Alveolar macrophage polarization at 7 days post-infection. (**B**) Interstitial macrophage polarization at 7 days post-infection. Data represent mean ± SEM from one representative experiment with four to seven mice per group; points show individual mice. Similar results were observed in two independent experiments. Two-way ANOVA with Tukey’s multiple comparison test: *P < 0.05; **P < 0.01; ***P < 0.001; and ns, not significant.

To further confirm the essential role of NLRC5 in TesG-induced macrophage polarization, we constructed *Nlrc5* knockout mice. Exons 1–8 of the *Nlrc5* gene were knocked out in C57BL/6 mice via CRISPR-Cas9 technology (Fig. S7A and B). Using chronic lung infection mouse models established with either PAO1 wild-type or Δ*tesG* mutant strains, we performed flow cytometry analysis of lung tissues collected at day 7 post-infection. The results demonstrated that the M2-type macrophage polarization triggered by TesG was eliminated in the alveolar macrophages of *Nlrc5* knockout mice compared to wild-type mice (Fig. 5A). A similar trend was also observed in interstitial macrophages (Fig. 5B), suggesting that TesG/NLRC5 signaling regulates macrophage polarization.

## DISCUSSION

*P. aeruginosa* can employ various strategies to alter the host immune microenvironment, promoting its immune evasion and long-term survival within the host. Our previous research found that the effector protein TesG can suppress the inflammatory response of the host, thereby promoting the development of the pathological process of chronic lung infection. Here, we demonstrate that TesG influences the polarization phenotype of macrophages through NLRC5 and further initiate a preliminary study on the regulatory mechanisms of macrophage function by TesG/NLRC5 signaling.

Macrophage polarization is the response of macrophages to different external stimuli and can be divided into classically activated M1-type macrophages and alternatively activated M2-type macrophages (37, 41–43). NLRC5 primarily functions as a negative regulator during host immune responses. However, there are no reports on its role in the regulation of macrophage polarization. Here, we show that NLRC5 is required for TesG-induced M2-type macrophage polarization. In particular, TesG/NLRC5 signaling in macrophage alternative activation appears to act through known NLRC5 regulatory pathways. Furthermore, we established chronic lung infection models in wild-type and *Nlrc5* knockout mice. TesG did not cause significant differences in cell polarization as observed in the wild-type group. Collectively, these findings clearly demonstrate that TesG promotes macrophage polarization toward the M2-type via NLRC5, aiding *P. aeruginosa* in evading immune surveillance.

Previous studies have indicated that the interaction between NLRC5 and target proteins may be direct or may require some auxiliary proteins for bridging (35). NAIP proteins serve as cytoplasmic receptors for a variety of bacterial protein ligands, capable of responding to a wide range of bacterial pathogens and mediating the recognition of pathogen patterns by NLRC4, which belongs to the same family as NLRC5 (38). In this study, we verified that there is no interaction between NLRC5 and TesG with or without NAIPs. However, this study still does not rule out the possibility that unknown proteins may mediate the interaction between TesG and NLRC5.

NLRC5 can inhibit NF-κB signaling by interacting with IKKα and IKKβ and blocking their phosphorylation, and it can inhibit IFN signaling by preventing MAV from binding to RIG-I and blocking the phosphorylation of IRF3 (33, 35, 40). Therefore, we examined whether TesG/NLRC5-induced macrophage polarization involved known NLRC5-associated pathways by using specific pathway inhibitors. Flow cytometry results showed that these inhibitors altered the proportion of macrophages polarized toward the M2 phenotype, supporting our hypothesis that TesG/NLRC5 signaling modulates these pathways to induce M2 polarization, but further investigation is necessary to delineate the precise mechanisms.

Based on our previous finding that TesG contributes to chronic *P. aeruginosa* infection, this study establishes the TesG/NLRC5 signaling axis as a key regulator of macrophage polarization, revealing a potential mechanism through which TesG facilitates bacterial immune evasion. Functional analysis of TesG-associated small GTPase targets showed that RhoA knockdown only modestly attenuated this response, suggesting possible functional redundancy among these proteins. Whether RhoA and NLRC5 are functionally linked remains to be investigated. Besides, several limitations should be acknowledged. While NLRC5 participates in inflammasome formation with NLRP3 (30), the effect of TesG on this process remains unclear. Moreover, while the subcellular localization of NLRC5 is critical for its function (44, 45), the detection of cytoplasmic and nuclear protein segregation with/without TesG treatment indicated that TesG does not affect the subcellular localization of NLRC5. Whether other possibilities exist also remains to be further elucidated. In summary, our report provides experimental evidence on the role of TesG in pathogen infections and suggests that NLRC5 is a potential biomarker for clinical anti-pathogen infection therapy.

## MATERIALS AND METHODS

### Bacterial strains and mammalian cell lines

The wild-type *P. aeruginosa* PAO1 strain (Harvard Medical School) served as the parental background; the Δ*tesG* mutant was generated in PAO1 by allelic exchange as described (12), selected by antibiotic and sacB/sucrose methods, and verified by junction PCR and Sanger sequencing. Strains were routinely cultured in Luria-Bertani broth with shaking (220 r.p.m.) at 37°C. Mouse immortalized bone marrow-derived macrophage iBMDMs were from Shao Feng’s laboratory. Mouse alveolar macrophage MH-S cells (ATCC CRL-2019) and HEK293T cells (ATCC CRL-3216) were obtained from the American Type Culture Collection. Cells were maintained in DMEM or RPMI-1640 (Gibco) supplemented with 10% FBS (Gibco).

### Mice

Eight-week-old female C57BL/6 mice were purchased from Beijing Huafukang Biotechnology Co., Ltd.; *Nlrc5* knockout mice were purchased from Saiye (Suzhou) Biotechnology Co., Ltd. Mice were housed in a specific pathogen-free facility of Sichuan University for breeding.

### Mouse models

For the chronic lung infection model, *P. aeruginosa* was embedded in agar beads and quantified as previously described (12, 34). The beads were diluted to 1–2 × 10^6^ CFUs in 50 µL sterile saline and intratracheally administered into anesthetized female C57BL/6 WT and *Nlrc5* knockout mice. On days 7 and 14 post-infection, lung tissues were collected, digested to generate single-cell suspensions, and subjected to flow cytometry to assess alveolar and interstitial macrophage populations.

### Preparation of single-cell suspensions from mouse lung tissues

Lung tissues from euthanized mice were excised and incubated in DMEM containing 5% collagenase I and IV for digestion. The tissues were mechanically dissociated, filtered through a 70 µm strainer, and centrifuged at 1,500 rpm for 5 min at 4°C. The pellet was treated with 10 mL erythrocyte lysis buffer on ice for 10 min, followed by two washes. The final pellet was resuspended in 1 mL of PBS to obtain a single-cell suspension for flow cytometry.

### Recombinant TesG protein

Recombinant TesG was prepared as described previously (12). Briefly, a codon-optimized PA4141/*tesG* was cloned into pET-32a via NcoI/HindIII, verified by restriction analysis and sequencing, and transformed into *Escherichia coli* BL21(DE3). His-tagged TesG expression was induced with IPTG, and the protein was purified by Ni-IDA affinity chromatography. Endotoxin was removed using ToxinEraser Endotoxin Removal Resin under cold conditions; LAL testing confirmed residual endotoxin <1 EU/μg for all preparations used.

### Cell treatment and phase-contrast imaging

iBMDMs or MH-S cells were seeded into 12-well plates and incubated overnight to allow adherence. The next day, cells were treated overnight as follows: IL-4/IL-13 (20 ng/mL each) as the positive control; heat-inactivated TesG (95°C, 10 min) as the negative control; and purified TesG at the indicated concentrations for experimental groups.

Phase-contrast images were captured from culture wells using an inverted microscope (ECLIPSE Ti2, Nikon) with a 20× objective. For each condition, at least three biological replicates were performed, and three randomly selected fields per replicate were imaged using identical acquisition settings.

### Inhibitor treatment *in vitro*

Adherent cells in 12-well plates were incubated overnight with TesG (10 µg/mL) together with DMSO (vehicle control) or in combination with one of the following inhibitors: Ac-YVAD-cmk (5 µM), peficitinib (1 µM), or IKK16 (0.1 µM) (all from MedChemExpress).

### siRNA transfection

Cells were transfected with gene-specific siRNAs (targeting NLRC5 or small GTPase family members) or a negative control siRNA. All siRNAs were synthesized by RiboBio. Transfections were performed at a final siRNA concentration of 50 nM using Xfect RNA Transfection Reagent (Takara) according to the manufacturer’s instructions. siRNAs used in subsequent experiments were selected and validated by qPCR. At 24 h post-transfection, cells were gently harvested and re-plated at equal density, allowed to re-adhere, and then subjected to the following TesG treatment.

### Quantitative PCR

Total RNA was extracted with TRIzol reagent (Thermo). Quantitative PCR was performed with the PrimeScript RT reagent Kit (Takara) and Green Premix Ex Taq II (Takara) on a LightCycler96 Real-time PCR System (Roche) following the manufacturer’s instructions. The qPCR primers for genes are listed in Table S1. The expression of GAPDH and β-actin was used as a control to calibrate the original mRNA concentrations in tissues and cells. Target gene expression was calculated via the 2^-ΔΔCT^ method.

### Flow cytometry

The single-cell suspensions from mouse lung tissues and cultured cells were prepared and counted, then stained with the following fluorescence staining antibodies from BioLegend: Mertk (151505, 1:100), F4/80 (123110, 1:100), CD206 (141720, 1:100), Siglec-F (155505, 1:100), CD11c (117306, 1:100), CD86 (105012, 1:100), and CD11b (101205, 1:100).

Samples were acquired on an Agilent NovoCyte flow cytometer using a consistent gating strategy, where the CD206^+^ population was defined by the negative control from the same experiment. Fluorescence compensation was performed with single-stained controls, and acquisition/compensation settings were held constant within experiments. All samples were run at matched cell concentrations.

### Co-immunoprecipitation assay

For co-immunoprecipitation, HEK293T cells were transfected with pMAX-Flag-TesG and pcDNA3.1-Myc-mNLRC5/hNLRC5. After 48 h, cells were lysed in IP buffer (Beyotime) with protease inhibitors (Roche). Lysates were incubated overnight at 4°C with anti-Myc magnetic beads (Thermo) or anti-Flag resin (BioLegend), followed by extensive washing and elution for Western blot analysis.

For endogenous pulldown, cell lysates were incubated overnight with anti-NLRC5 (LifeSpan BioSciences) or anti-TesG antibody (TesG polyclonal antibody obtained from TesG-immunized rabbits) ± recombinant TesG protein. Protein A/G agarose beads (Beyotime) were then added and incubated for 4 h. After washing, bound proteins were analyzed by SDS-PAGE.

### Western blot analysis

Proteins were extracted using RIPA buffer, separated by SDS-PAGE, and then transferred to PVDF membranes (Millipore). Membranes were blocked with 5% skim milk at 37°C for 1 h, incubated with primary antibodies overnight at 4°C, and then incubated with HRP-labeled secondary antibodies. Signals were detected using ECL (Beyotime) and imaged with an iBright CL1500 (Thermo). Primary antibodies included anti-NLRC5 (LifeSpan BioSciences), anti-GAPDH (Cell Signaling Technology), anti-FLAG (MBL), and anti-Myc (Cell Signaling Technology).

### Statistical analysis

The experimental data were plotted and statistically analyzed using GraphPad Prism 10. Results were expressed as the mean values ± standard error of the mean; unpaired two-tailed Student’s *t* test was used to compare two groups of data; comparisons between more than two groups were performed by one-way ANOVA or two-way ANOVA (with Tukey’s multiple comparison test), and differences were considered to be statistically significant at *P* < 0.05 (*P < 0.05, ** *P* < 0.01, *** *P* < 0.001, and **** *P* < 0.0001).

## Data Availability

The raw data generated in this study correspond to the Agilent Mouse microarray outputs. These data have been deposited in the NCBI BioProject under accession number PRJNA1196168 and are publicly available at: https://www.ncbi.nlm.nih.gov/bioproject/PRJNA1196168.

## SUPPLEMENTAL MATERIAL

Supplemental figures msphere.00681-25-s0001.pdf Figures S1-S7.

Table S1 msphere.00681-25-s0002.xlsx Strains, plasmids, and primers.

ASM does not own the copyrights to Supplemental Material that may be linked to, or accessed through, an article. The authors have granted ASM a non-exclusive, world-wide license to publish the Supplemental Material files. Please contact the corresponding author directly for reuse.
