# Supplementary material for: The Pseudomonas aeruginosa effector protein TesG regulates alternative activation of macrophages through NLRC5
Source: mSphere. 2025 Oct 31;10(11):e00681-25. doi: 10.1128/msphere.00681-25 (PMC12646011; doi:10.1128/msphere.00681-25)
Supplement: Supplemental figures — Figures S1-S7. [file msphere.00681-25-s0001.pdf]

Supplementary Information of

The *Pseudomonas aeruginosa* effector protein TesG regulates alternative activation of macrophages through NLRC5

Qianhua Zhang[1,#], Yige Zhang[1,#], Ruihuan Wang[1,#], Kailun Wang[1,#], Teng Ma[1], Chaoyu Zou[1], Yongxin Zhang[1], Xueli Hu[1], Huan Liu[1], Jing Sherly Li[1], Yang Yang[1], Zhuochong Liu[1], Miao Tang[1], Yilin Liu[1], Hongliang Li[1], Yu Tang[1], Jing Li[2], Xikun Zhou[1,*]

[1]Department of Biotherapy, Cancer Center and State Key Laboratory of Biotherapy, West China Hospital, Sichuan University, Chengdu 610041, China.

[2]State Key Laboratory of Oral Diseases, National Clinical Research Center for Oral Diseases, Chinese Academy of Medical Sciences Research Unit of Oral Carcinogenesis and Management, West China Hospital of Stomatology, Sichuan University, Chengdu, China.

Running head: TesG regulates macrophage activation via NLRC5

[#]These authors contributed equally: Qianhua Zhang, Yige Zhang, Ruihuan Wang and Kailun Wang

[*]Correspondence: xikunzhou@scu.edu.cn

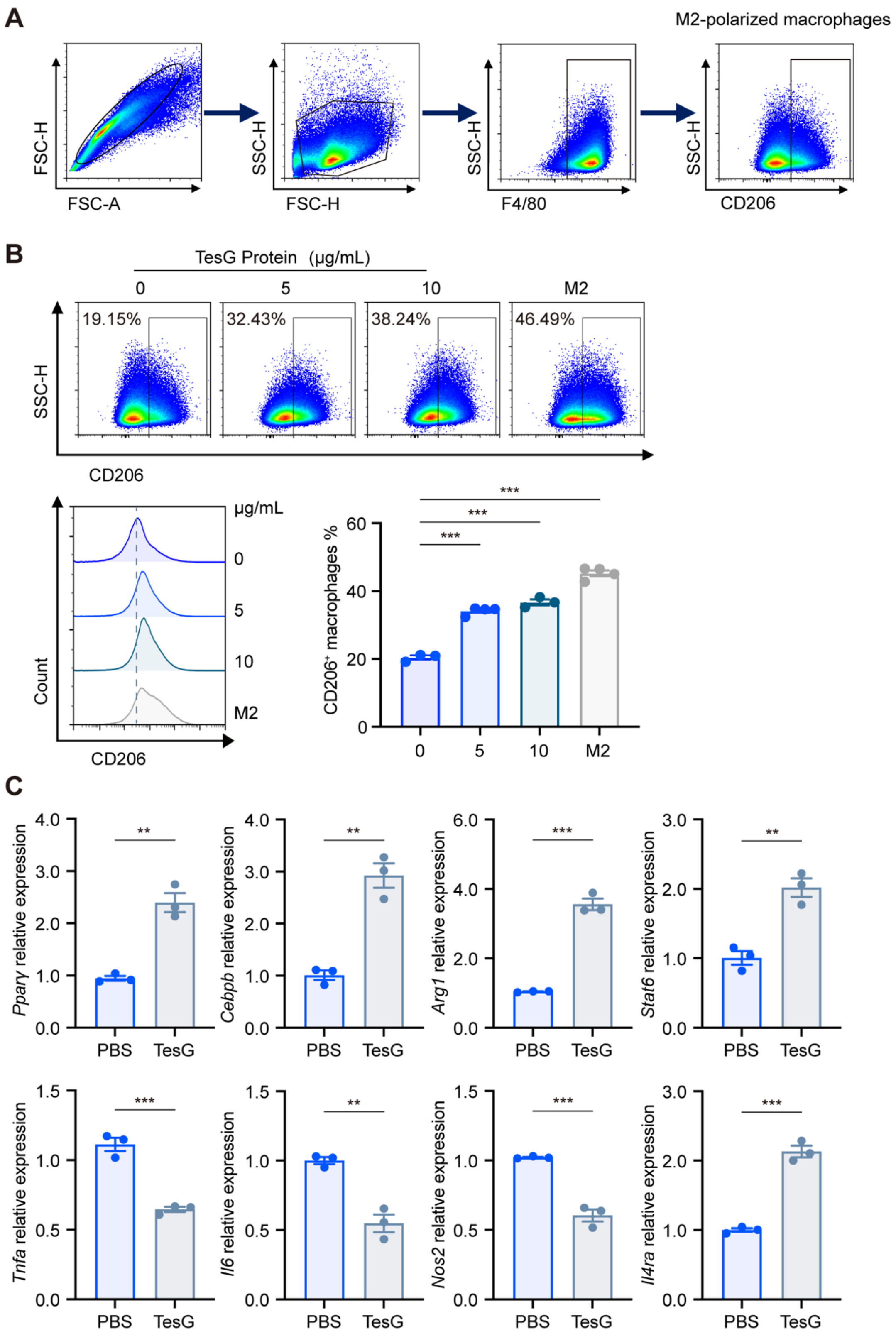

Figure S1 | TesG induces the polarization of MH-S cells. A Gating strategy for identification of M2-polarized macrophages. Live single cells were first gated on F4/80^{+} macrophages, followed by selection of CD206^{+} cells within this population to identify M2-polarized macrophages. **B** Proportion of CD206^{+} MH-S cells after overnight treatment. IL-4/IL-13 (20

ng/mL each) served as M2 positive control (n = 3 or 4). **C** Relative mRNA expression levels of polarization-related genes in TesG-treated MH-S cells (n =3). Data points represent biological replicates within individual experiments; bars show mean ± SEM. Similar results were observed in three independent experiments. *P < 0.05; **P < 0.01; ***P < 0.001; ns, not significant (**B**: one-way ANOVA with Tukey's multiple comparison test; **C**: two-tailed unpaired Student's t test).

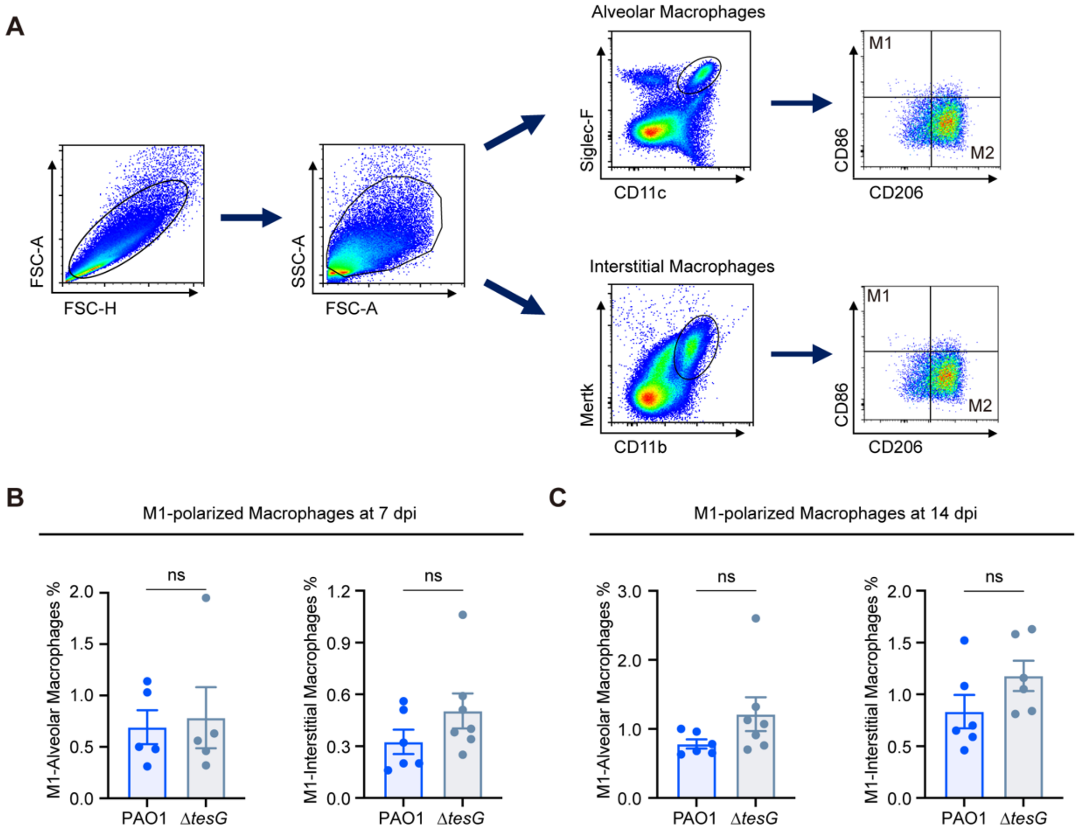

Figure S2 | Gating strategy of alveolar macrophages and interstitial macrophages in flow cytometry analysis related to Fig. 2. A Macrophage subpopulations were identified by flow cytometry: alveolar macrophages ($CD11c^{+}$Siglec-F^{+}), interstitial macrophages ($CD11b^{+}Mertk^{+}$), M1-polarized ($CD86^{+}$), and M2-polarized ($CD206^{+}$) populations. **B, C** Polarization of alveolar macrophages and interstitial macrophages *in vivo* following chronic infection with PAO1 and Δ*tesG* strains at 7 dpi (**B**) and 14 dpi (**C**). Data are from one experiment with 5-7 mice per group; points represent individual mice, bars show mean ± SEM. Similar results were observed in two independent experiments. Statistical significance was calculated using an unpaired t test: no significant difference (ns).

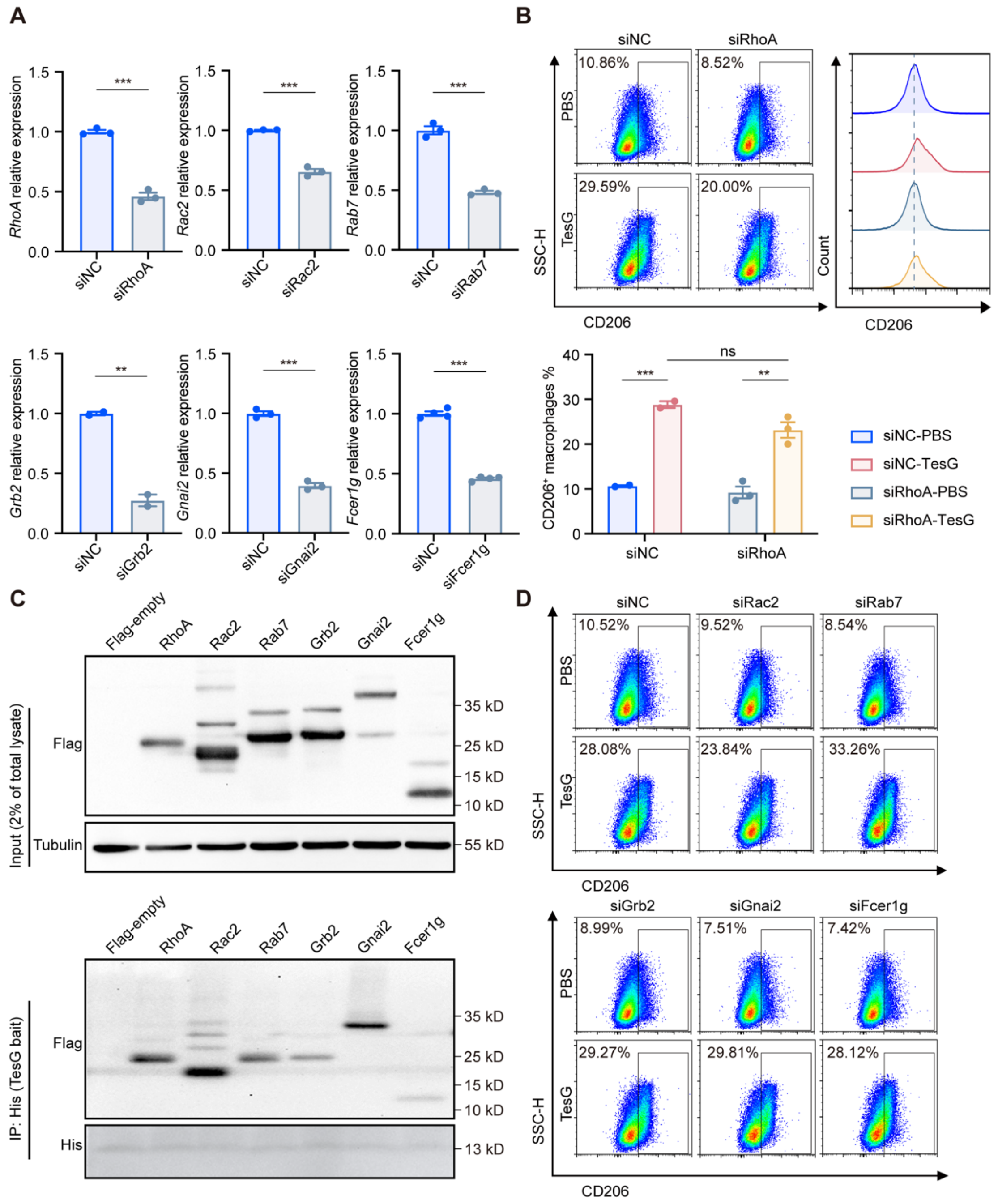

Figure S3 | Immune interacting proteins weakly influence TesG-induced the polarization of macrophages. A siRNAs targeting TesG-associated immune interactors were designed and validated for knockdown efficiency. **B** TesG-induced macrophage polarization following *RhoA* interference in MH-S cells. **C** Validation of TesG interactions with immune-associated proteins by co-immunoprecipitation. His-TesG served as bait protein (IP: His); co-precipitated Flag-tagged candidate proteins (RhoA, Rac2, Rab7, Grb2, Gnai2, Fcer1g) were detected with anti-Flag antibody. Flag-empty vector was included as negative control. **D** Impact of immune-interacting gene interference on TesG-induced macrophage polarization. Flow cytometry assay

was performed after overnight treatment with TesG (10 µg/mL). Data points represent biological replicates within individual experiments; bars show mean ± SEM. Statistical significance: $^{*}P < 0.05$; $^{**}P < 0.01$; $^{***}P < 0.001$; ns, not significant (**A**: two-tailed unpaired Student's t test; **B**: two-way ANOVA with Tukey's multiple comparison test).

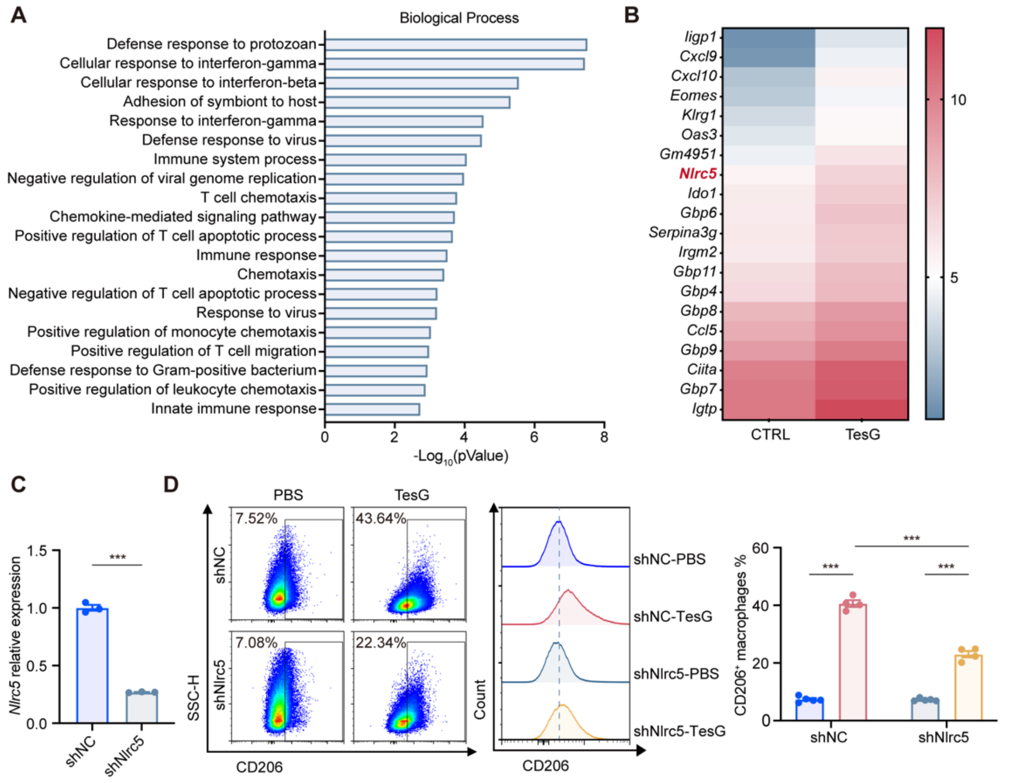

Figure S4 | NLRC5 is involved in TesG-induced the polarization of macrophages. 20 µg of TesG protein was injected into each mouse by tail vein injection every other day for a total of 3 injections, and mouse lung tissues were harvested 24 h after the last injection of TesG protein for whole transcriptome microarray assay. **A** GO biological process enrichment of differentially expressed genes. Top 20 significant pathways are shown. **B** Heatmap of immune-related gene expression profiles ($|\log_2 FC| > 1$, $P < 0.05$). The heatmap color scale represents normalized expression values. **C** shRNA-mediated *Nlrc5* silencing was verified by qPCR. **D** Polarization typing of iBMDM macrophages after *Nlrc5* interference. All experiments included 3-5 biological replicates, all data are presented as the mean ± SEM. Statistical significance: $^*P < 0.05$; $^{**}P < 0.01$; $^{***}P < 0.001$; ns, not significant (**C**: two-tailed unpaired Student's t test; **D**: two-way ANOVA with Tukey's multiple comparison test).

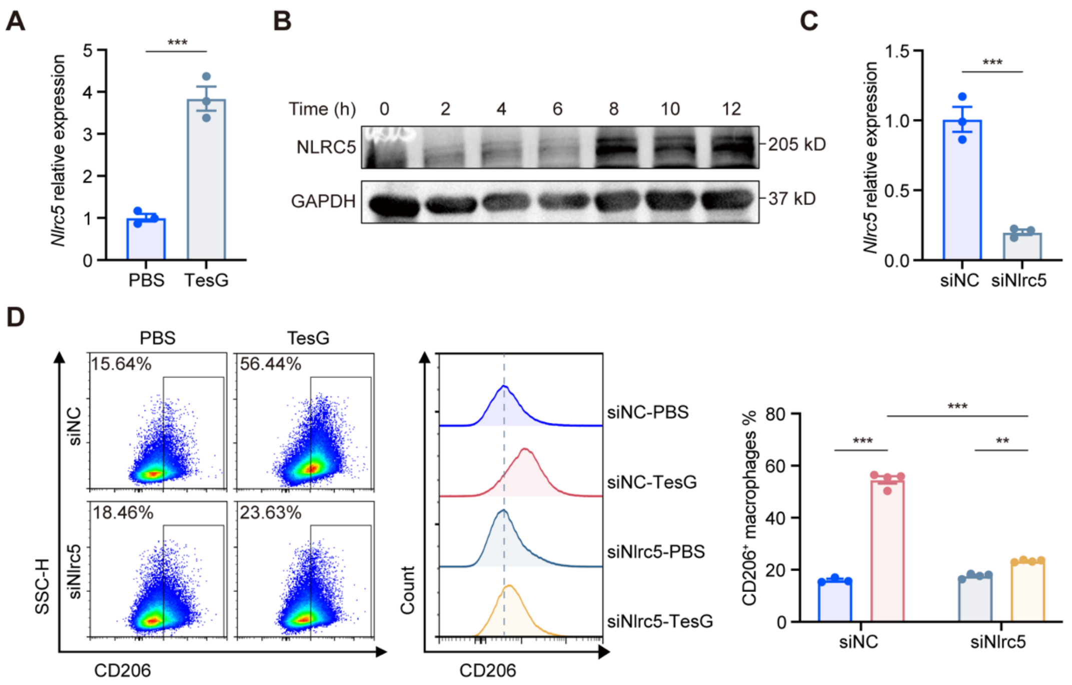

Figure S5 | NLRC5 in MH-S cells is involved in TesG-induced the polarization of macrophages. A The mRNA expression level of *Nlrc5* was upregulated in MH-S cells following overnight treatment with 10 μg/mL TesG protein. **B** Western blot analysis demonstrates TesG progressively induced NLRC5 protein expression. **C** Interference efficiency of *Nlrc5* siRNA in MH-S cells. **D** Flow cytometry analysis of macrophage polarization following TesG treatment in *Nlrc5*-knockdown MH-S cells. All experiments included 3-4 biological replicates, all data are presented as the mean ± SEM. Statistical significance: **P* < 0.05; ***P* < 0.01; ****P* < 0.001; ns, not significant (**A**, **C**: two-tailed unpaired Student's t test; **D**: two-way ANOVA with Tukey's multiple comparison test).

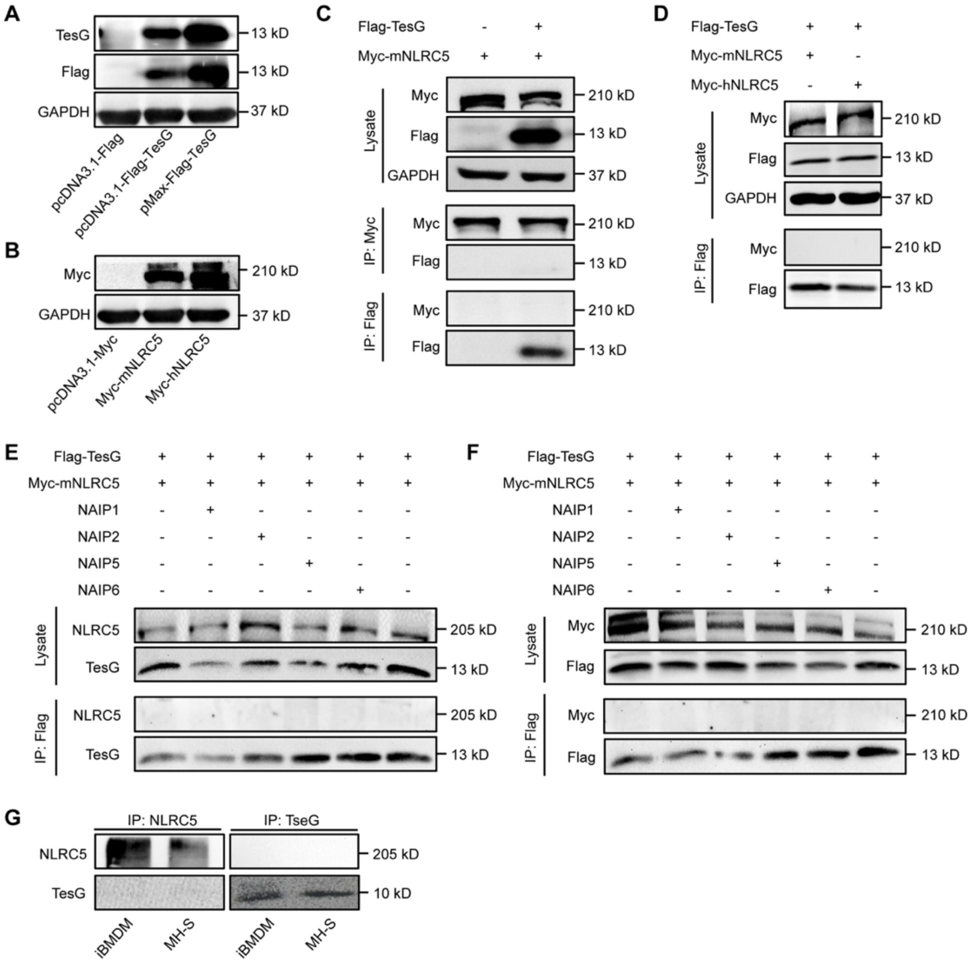

Figure S6 | TesG does not directly interact with NLRC5. A Western blot validation of TesG overexpression in 293T cells transfected with either pcDNA3.1-Flag-TesG or pMAX-Flag-TesG constructs. **B** Western blot analysis of human (Myc-hNLRC5) and murine (Myc-mNlrc5) NLRC5 overexpression in 293T cells. **C, D** Co-immunoprecipitation analysis of TesG-NLRC5 interaction. 293T cells were co-transfected with Flag-TesG and Myc-mNLRC5. Immunoprecipitation was performed using either anti-Myc magnetic beads or anti-Flag resin (**C**). No direct interaction was detected between TesG and either mNLRC5 or hNLRC5 (**D**). **E, F** Co-IP results of TesG and NLRC5 in NAIP-mediated situations. TesG, NLRC5 and NAIP overexpression vectors were cotransfected into 293T cells, and interactions were detected with protein antibodies (**E**) and labeled antibodies (**F**). **G** Endogenous NLRC5 pull-down assays were performed using two types of macrophages, followed by co-incubation with recombinant TesG proteins *in vitro*. No interaction between TesG and NLRC5 was detected.

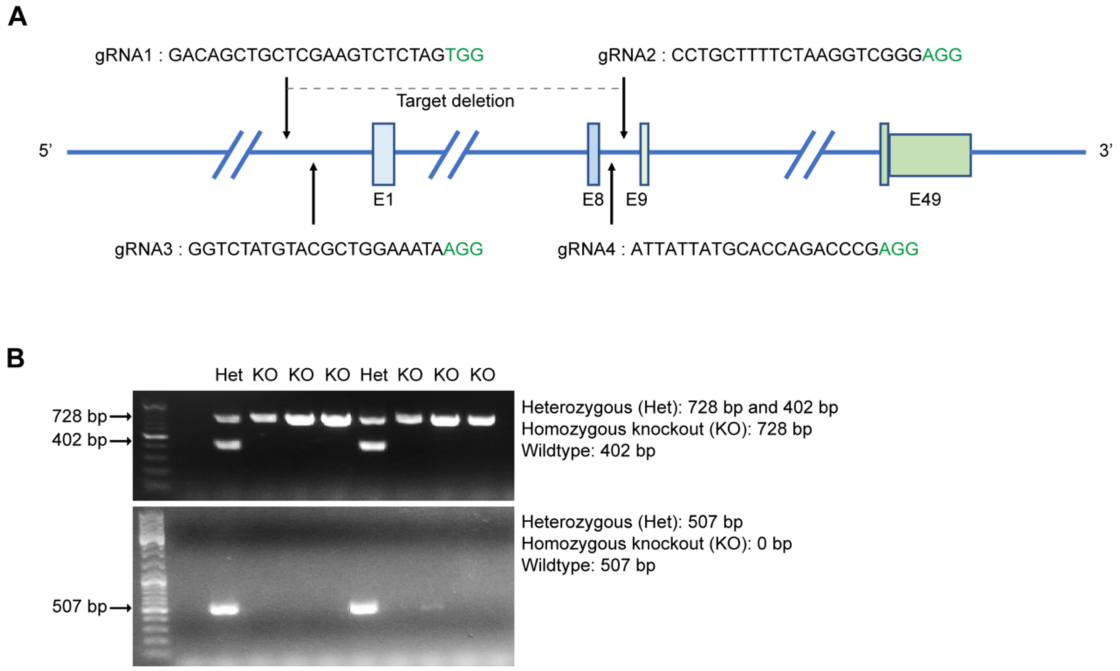

Figure S7 | Construction of the *Nlrc5* knockout mice. A Principle of *Nlrc5* knockout mouse construction. Silencing of *Nlrc5* expression by knocking out exons 1-8 of the *Nlrc5* gene in C57BL/6 mice by CRISPR-Cas9 technology. **B** Genotyping verification of *Nlrc5* knockout mice. Representative PCR result using two primer sets demonstrates: 2 heterozygous (Het) mice and 6 homozygous (KO) mice.
